# Synchronous precipitation reduction in the American Tropics associated with Heinrich 2

**DOI:** 10.1038/s41598-017-11742-8

**Published:** 2017-09-11

**Authors:** Martín Medina-Elizalde, Stephen J. Burns, Josué Polanco-Martinez, Fernanda Lases-Hernández, Raymond Bradley, Hao-Cheng Wang, Chuan-Chou Shen

**Affiliations:** 1 Department of Geosciences, Auburn University, Alabama, USA; 2 Department of Geosciences, University of Massachusetts, Amherst, MA USA; 3 Basque Centre for Climate Change (BC3), Leioa, Bizkaia Spain; 4 UMR CNRS 5805 EPOC, University of Bordeaux, 33615, Pessac, France; 5 Laboratoire Paleoclimatologie et Paleoenvironnements Marins, EPHE, PSL Research University, Pessac, France; 6 Department of Earth Sciences, National Autonomous University of Mexico (UNAM), D.F., Mexico; 7 High-Precision Mass Spectrometry and Environment Change Laboratory (HISPEC), Department of Geosciences, National Taiwan University, Taipei, Taiwan, ROC

## Abstract

During the last ice age temperature in the North Atlantic oscillated in cycles known as Dansgaard-Oeschger (D-O) events. The magnitude of Caribbean hydroclimate change associated with D-O variability and particularly with stadial intervals, remains poorly constrained by paleoclimate records. We present a 3.3 thousand-year long stalagmite δ^18^O record from the Yucatan Peninsula (YP) that spans the interval between 26.5 and 23.2 thousand years before present. We estimate quantitative precipitation variability and the high resolution and dating accuracy of this record allow us to investigate how rainfall in the region responds to D-O events. Quantitative precipitation estimates are based on observed regional amount effect variability, last glacial paleotemperature records, and estimates of the last glacial oxygen isotopic composition of precipitation based on global circulation models (GCMs). The new precipitation record suggests significant low latitude hydrological responses to internal modes of climate variability and supports a role of Caribbean hydroclimate in helping Atlantic Meridional Overturning Circulation recovery during D-O events. Significant in-phase precipitation reduction across the equator in the tropical Americas associated with Heinrich event 2 is suggested by available speleothem oxygen isotope records.

## Introduction

During the last ice age climate in the North Atlantic oscillated on millennial time scales from cold (stadial) to warm (interstadial) episodes, often referred to as Dansgaard-Oeschger (D-O) events^1, 2^. These D-O events were first identified in Greenland ice cores but are expressed in terrestrial lakes, speleothems, and marine sediments from the North Atlantic, South America, Pacific and Indian Oceans^3–8^. The leading hypothesis to explain millennial-scale climate cycles invokes changes in the Atlantic Meridional Overturning Circulation (AMOC)^9^. At times, during the last glacial, the Northern Hemisphere ice sheets supplied sufficient freshwater to the North Atlantic to reduce AMOC by lowering surface ocean density^10^. In turn, AMOC slowdown decreased heat transport from low to high latitudes leading to cooling across the North Atlantic^9^. Meltwater pulses are reflected by episodes of thick ice rafted debris (IRD) deposits in the North Atlantic known as Heinrich (H) events that were generally associated with stadial episodes^11–13^.

There is increasing evidence that D-O cycles were associated with rapid and significant hydrological responses in tropical and subtropical regions worldwide, probably linked to the position of the Intertropical Convergence Zone (ITCZ) and its belt of convective activity. Paleoclimate records, particularly, suggest hydrological variability that is out-of-phase across the equator in the Atlantic and eastern Pacific regions on millennial time scales^7, 8, 14, 15^ consistent with model results of AMOC slowdown^16, 17^. AMOC slowdown via freshwater forcing creates a thermal asymmetry across the equator that leads to a southward displacement of the ITCZ in the Atlantic and Pacific, which then enhances precipitation in South America and the opposite in North America and northern South America, such as the Cariaco Basin^8, 16–19^. Model results suggest, furthermore, that AMOC slowdown can induce intense precipitation reductions in the North Atlantic and India of up to 80%^16^. The frequency and magnitude of tropical hydroclimate variability during times of AMOC reduction remain, however, largely unconstrained by paleoclimate records. We present a last glacial stalagmite δ^18^O record of Caribbean climate from the Yucatan Peninsula (YP), Mexico, and attempt to reconstruct quantitative precipitation variability to establish how rainfall in the region responds to D-O events and particularly during stadial intervals. Our precipitation estimates are based on an assessment of last glacial tropical temperatures^31^, shifts in the δ^18^O composition of precipitation suggested by global circulation models (GCMs), the observed regional amount effect^23^, and the assumption that temperature dependence fractionation during calcite precipitation was according to the model by Tremaine *et al*.^32^ (more details in Methods). We acknowledge that this is a first attempt to estimate last glacial precipitation changes quantitatively and that uncertainties still remain, particularly, concerning the magnitude of the amount effect and the δ^18^O composition of precipitation, and biases due to potential calcite isotopic disequilibrium conditions, during this time interval. The new precipitation record spans the time between 26.4 and 23.2 ka BP, corresponding to stadial 2 (S2) in the North Greenland Ice Core Project (NGRIP) δ^18^O record^20^ and covers the full duration of Heinrich event 2 (H2).

## Results and Discussion

**Figure 1:**
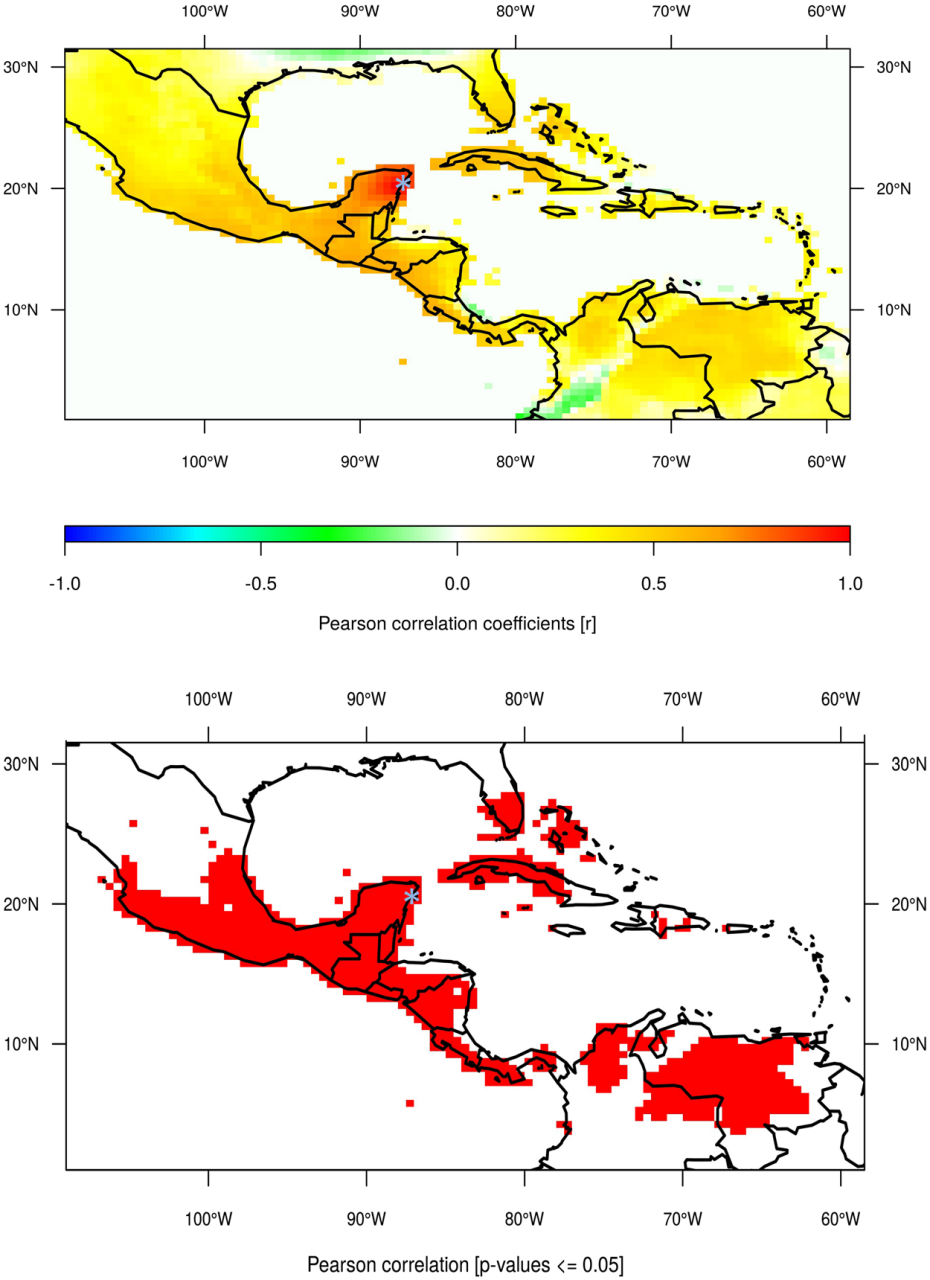
Spatio-temporal correlation analysis of precipitation (monthly values from 01/1901 to 12/2013 and with a spatial coverage of 0.5° latitude by 0.5° longitude) at the location (20°15′N, 87°45′W) (close to Playa del Carmen, Yucatan, Mexico). Location of Rio Secreto indicated with light blue asterisk. The precipitation data set comes from the GPCC Global Precipitation Climatology Centre and is available from https://www.esrl.noaa.gov/psd/data/gridded/data.gpcc.html. The map was created using the R software^72^.

In 2012, we retrieved a 87-cm long stalagmite specimen, named Itzamna (after the Maya god of creation), from Río Secreto Natural Reserve, a spectacular semi-inundated cave system located in Playa del Carmen City, in the Caribbean coast of the YP (20°35.244′N; 87°8.042′W) (Supplementary Fig. S1). The YP, located between the latitudes 18 and 21°N, experiences a very distinctive monsoonal precipitation cycle with up to 80% of annual precipitation falling between June and October^21^ (Fig. S2). The dominant moisture source of the YP is the Caribbean Sea^22, 23^ and interannual precipitation in this broad region is significantly correlated with precipitation in the YP (Fig. 1).

The Itzamna stalagmite time scale for the oldest section was determined with 7 absolute U-Th dates, following methods by^24–26^ and based on Montecarlo simulations using the COPRA software^27^(Table S2; Methods). This stalagmite first deposited from 26.5 ka BP to 23.2 ka BP. After a long interruption, it resumed growth at BCE 1037 and stopped growing at CE 398. The younger portion of the stalagmite that spans the interval from the Middle Preclassic Period to the Early Classic Period of Maya history (BCE 1000 to CE 400) has been published previously^23^. This study focuses on the stalagmite’s oldest section.

**Figure 2:**
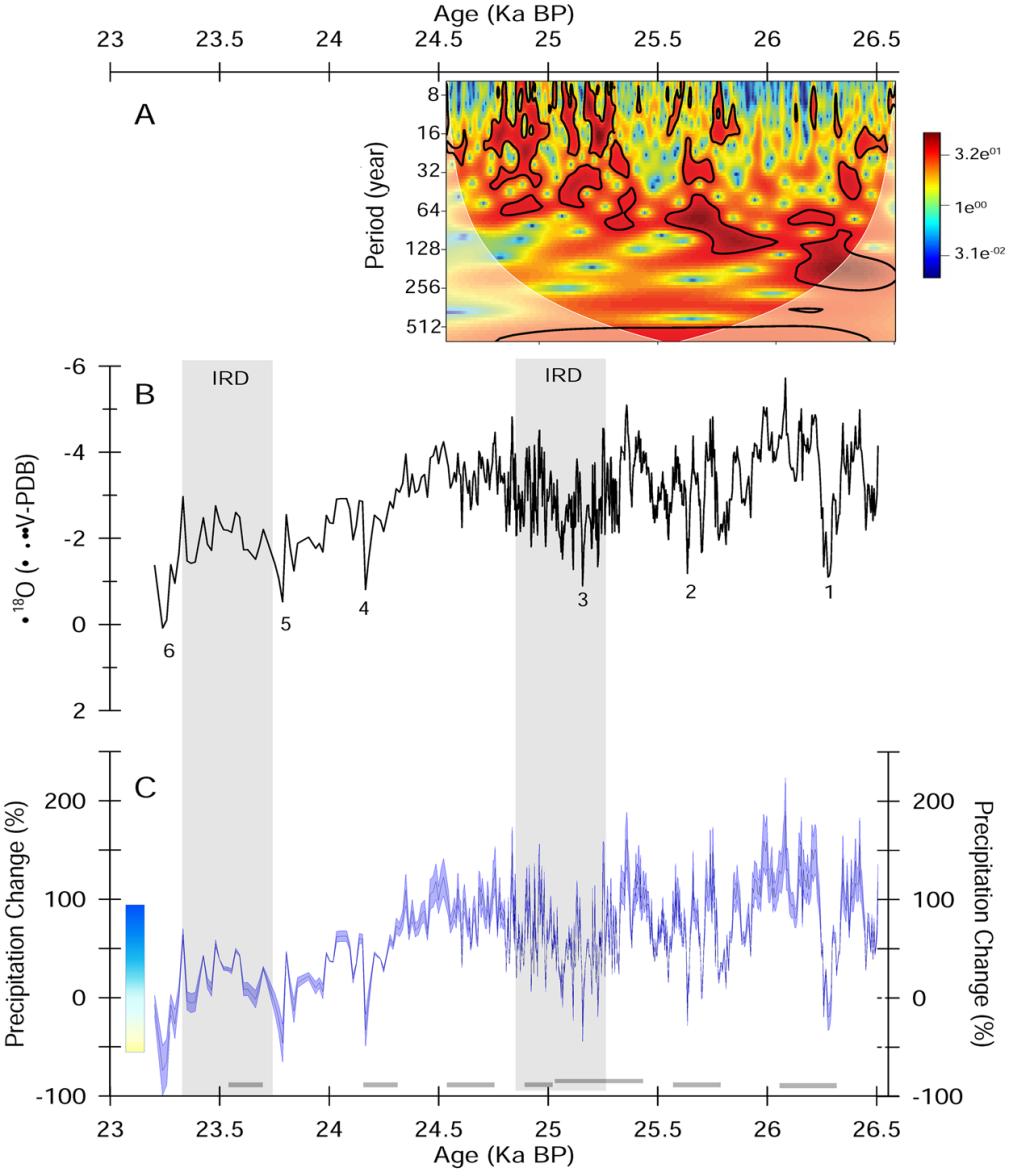
Itzamna δ^18^O (panel B) and δ^18^O-derived precipitation (panel C) records spanning the time interval between 26.4 and 23.2 ka BP from Rio Secreto, Playa del Carmen, Mexico. Precipitation percent change estimates are relative to the mean annual precipitation amount in Playa del Carmen today of 1465 mm (panel B). These records have a resolution during the time interval between 26.5 and 24.6 ka BP of 3.4 ± 1.3 years (1 SD) and between 24.6 and 23.2 ka BP of 16 ± 6.7 years (1 SD). Precipitation change estimates (blue shading) reflect reference values of calcite precipitated under isotopic equilibrium conditions with last glacial dripwater temperature (18–20 °C) and an amount effect (*δP*/*ΔP*) ranging from −0.0168‰ per mm to −0.0106‰ per mm, corresponding to the 80% confidence interval of the amount effect slope based on instrumental data (see Methods section for more details). Average precipitation change is indicated by the blue the line within blue shading. Reference equilibrium values were calculated using the paleotemperature equation by ref. 32. The potential bias in our precipitation estimates due to shifts in annual precipitation amount seasonality is ±8% in the range of 55 to 70% summer contribution to annual precipitation (see Methods section). Vertical box on the left of panel B; indicates precipitation variability observed today in the northeast of the YP (mean = 1390 mm per year; range 670 mm to 2664 mm per year), based on the longest continuous instrumental record of precipitation available for this region (i.e. Kantunilkil Station)^21^. The record covers Stadial 2 and Heinrich event 2 (H2). Vertical grey bars reflect the two peak IRD depositional phases associated with H2, centered at 25 ± 0.2 ka BP and 23.5 ± 0.2 ka BP^13^. The six significant positive δ^18^O shifts discussed in the manuscript are indicated with numbers. Top image (panel A), represents a wavelet spectral power analysis of the Itzamna δ^18^O record. The area outside the white lines indicates the cone of influence where the edge effects of the wavelet transform and uncertainties become important and the black contours reflect statistically significant power spectra (CI = 95%). Wavelet spectral power is based on methods by refs 73, 74, as implemented in the R package “biwavelet”^72^.

The Itzamna δ^18^O record (n = 647 isotopic analyses at 0.5 mm sampling resolution) has a resolution of 3 ± 1 years (1 SD) during the time interval between 26.5 and 24.6 ka BP and of 16 ± 6.7 years (1 SD) between 24.6 and 23.2 ka BP (Fig. 2B). Calcite δ^18^O analyses are reported relative to V-PDB and reproducibility of the standard materials is better than 0.1‰ (more details in Methods). The stalagmite δ^18^O record from 26.5 to ~24.5 ka BP shows no trend. At ~24.5 ka stalagmite δ^18^O values become progressively more positive with a statistically significant slope of −1.7‰ kyr^−1^ (R^2^ = 0.5, p < 0.01). This positive δ^18^O trend is associated with a decrease in stalagmite growth rates (Fig. S3). Across the length of the record there are six significant positive δ^18^O shifts (2 SD from the record’s mean) of relatively short duration, 1–2 decades (Fig. 2, positive shifts indicated with numbers). The three most recent shifts are associated with a positive trend that culminates in the most positive short term shift in stalagmite δ^18^O at 23.24 ka BP, preceding the growth hiatus mentioned above (Fig. 2).

The characteristics and physical conditions of the Río Secreto cave system monitored from the year 2014–2016 (relative humidity ~100%, stable temperature and dripwater δ^18^O composition), a Hendy test (Fig. S4) and isotopic equilibrium calculations (e.g. based on dripwater δ^18^O and temperature and fresh calcite)^28^ suggest that Itzamna’s calcite was precipitated under or near isotopic equilibrium conditions and faithfully records precipitation δ^18^O variability^23^ (see Methods for more details). Precipitation δ^18^O in tropical regions reflects an amount effect on interannual^22^ and seasonal timescales^29^, as confirmed by our instrumental data for the YP (2014–2016)^23^.

We estimate precipitation percent changes relative to modern precipitation amount conditions following previous protocols^30^. Briefly, to estimate precipitation changes, we shift the annual precipitation cycle in Playa del Carmen (5-year monthly averages, 1998–2003) and thus precipitation δ^18^O using the local amount effect, so that the annual amount-weighted δ^18^O of precipitation varies in accordance with Itzamna δ^18^O anomalies^23, 30^ (Fig. 2 and Table S3). Itzamna δ^18^O anomalies are calculated relative to the expected equilibrium δ^18^O composition of calcite associated with the annual precipitation amount cycle in Playa del Carmen today (1465 mm/year), after adjusting precipitation δ^18^O and dripwater temperatures to reflect last glacial conditions (see Methods). The full range of stalagmite δ^18^O-derived precipitation values are represented by combinations of estimates of last glacial regional surface temperature (from 18 °C to 20 °C)^31^ and modern instrumentally-determined amount effect slopes (*δP*/*ΔP* = *−*0.0106‰/mm and −0.0168‰/mm) (see Methods; Fig. 2C).

The Itzamna δ^18^O record suggests that precipitation in the northeastern YP during S2 varied typically between +150% and −10% (72 ± 80%, 2 SD) relative to today (i.e. 1465 mm/year) similar to the regional interannual precipitation range observed today but exceeding the modern upper boundary suggested by the longest continuous instrumental record of precipitation (Kantunilkin Town, 1950–2011, +92% to −51%)^23^ (Fig. 2C and Table S3). Based on average precipitation estimates, during the three most recent time intervals of extreme precipitation reduction, between 23.3–23.7 ka BP and centered at 24.1 and 25 ka BP, annual mean precipitation was reduced to a minimum of 395–980 mm (Fig. 2). This annual precipitation amount range can be found occurring today in the northern YP^21^ (Fig. S5). Notably, the intense precipitation reductions between 23.2–23.7 ka BP and centered at 25 ka BP, coincide with two peak IRD depositional phases associated with H2 centered at 25 and 23.5 ka BP^13^ (Fig. 2 horizontal gray bars).

The Itzamna δ^18^O-precipitation record shows significant variability on interannual and multidecadal time scales. Particularly, wavelet spectral power analysis indicates significant variability centered at periods of ~8, ~16, ~32, ~64 and ~128 years, over the first 2 kyrs of the record (Fig. 2A). We find that chronological uncertainties have little effect on these results (see Methods for more details). The record shows variability only at the higher periods from 64 to 128 years after 24.5 ka because stalagmite growth rate reduction at this time causes insufficient resolution to detect higher frequency δ^18^O variability (Fig. S6).

Itzamna precipitation record variability on multidecadal time scales, 32–64 years, may reflect a potential connection with the dominant pattern of North Atlantic sea surface temperature (SST) associated with the Atlantic Multidecadal Oscillation (AMO)^33–35^. Instrumental, paleoclimate and modeling data support a link between AMO and hydroclimate variability over large parts of the North Atlantic^35, 36^, African Sahel^37, 38^, western Europe^36, 39, 40^, northeastern Brazil^37^, Cuba^41^ and North America^42, 43^. Particularly for the Gulf of Mexico and Caribbean regions, model simulations and analyses of instrumental data suggest a positive correlation between precipitation and the phases of AMO^36, 44^. Large-scale hydrological responses to AMO have been associated with variations in the SST gradient between the north and south Atlantic^40, 45^, shifts in the mean Intertropical convergence zone (ITCZ)^36^ and changes in Atlantic tropical cyclogenesis^36, 46^.

Higher frequency variability at an ~8 year period in the Itzamna precipitation record may reflect El Niño-Southern Oscillation (ENSO) modulation of Caribbean hydroclimate (Fig. 2A). Studies with models and historical records suggest that Caribbean precipitation regimes are modulated by ENSO^47–49^ and there is evidence that this modulation could be via the influence of ENSO on tropical Atlantic cyclogenesis^23, 50^. Atlantic hurricanes are suppressed when El Niño conditions in the Pacific induce upper altitude increased wind shear in the Atlantic, thus reducing rainfall fluxes over the YP by tropical cyclones^51, 52^.

Interannual and multidecadal variability (~8–128 year periods) is also suggested by four other stalagmite δ^18^O records spanning the Late Holocene from the YP^23, 53^, Belize^50, 54^ and Cuba^41^ and by last glacial lacustrine paleoenvironmental records from Lake Petén Itzá^6, 55, 56^. Observed and inferred hydrological change in the Caribbean and Gulf of Mexico regions during the Holocene and last glacial intervals indicate that Atlantic low latitude regions respond to internal modes of climate variability on interannual (i.e. ENSO) and multidecadal time scales (i.e. AMO) regardless of the global climate state (i.e. interglacial versus glacial conditions).

**Figure 3:**
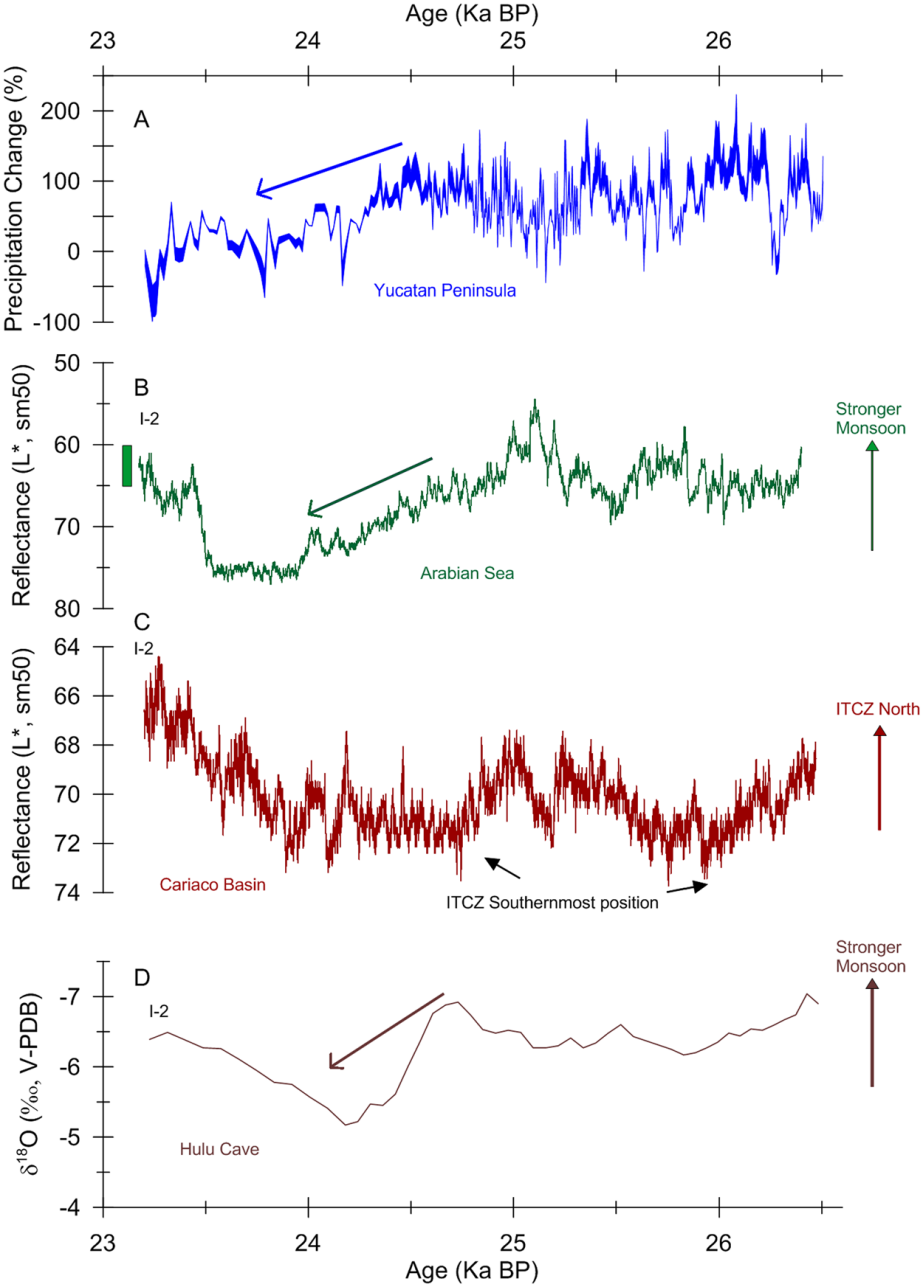
(**A**) Itzamna stalagmite precipitation record (this study), (**B**) northeastern Arabian sea sediment reflectance L* record^8^ and (**C**) Cariaco Basin, northern Venezuela, sediment reflectance L* record^8^. Arrows illustrate weakening of YP precipitation and Indian monsoon. Green vertical bar in panel B represents the Arabian Sea sediment reflectance range reflecting Holocene values. This range illustrates that the intensity of the Indian Monsoon was not significantly different in the early stages of S2 than during the Holocene. For reference, Cariaco Basin sediment L* reflectance values associated with previous stadial episodes (e.g. stadials 3–18) were lower than the lowest S2 values, thus suggesting that the Atlantic ITCZ was not particularly displaced south during S2 relative to a typical stadial. Differences in the timing of interstadial 2 among these records reflect chronological uncertainties >200 years^4, 8^.

**Figure 4:**
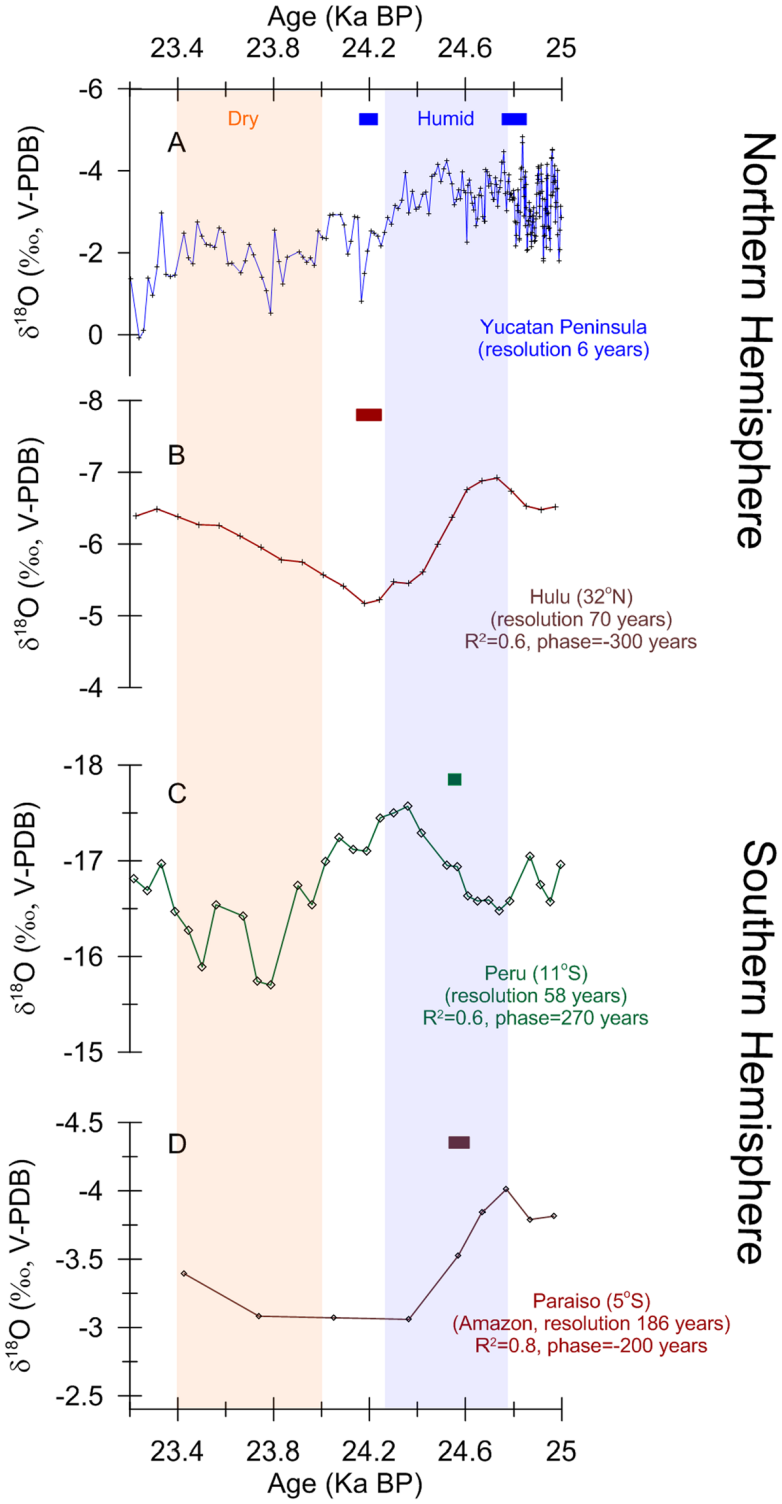
Blow up comparing speleothem δ^18^O records from the northern and the southern Hemispheres between 25 and 23.2 ka BP. Speleothem δ^18^O record from the Yucatan Peninsula (20°N) (**A**), Hulu, China (32°N)^4^ (**B**), Perú (11°S)^7^ (**C**), and Paraíso, Amazon Basin (5°S)^15^ (**D**). The time resolution of these records is shown. Cross-correlation coefficient (r^2^) and phase relationship between the YP speleothem δ^18^O record and the other speleothem δ^18^O records are shown. Negative/positive phase values represent that the YP δ^18^O record lags/leads the other speleothem δ^18^O records, respectively. These records are in phase within their combined age uncertainties. Small color box above each record shows the U-Th age and its uncertainty (2 Sigma) available for each record within this time interval (Itzamna = ±73 years; Hulu = ±100 years; Perú = ±50 years; Paraíso = ±84 years). Vertical color bars illustrate the transition from humid to drier conditions as suggested by these speleothem δ^18^O records reflecting precipitation amount.

As previously described, the Itzamna precipitation record suggests a significant long-term drying trend that began approximately 24.5 ka BP, following estimated precipitation mean maximum levels of + 150% and humid conditions in Central America^6, 55^. This drying trend ended about 23.2 ky ago when mean precipitation declined by about 50% (based on the average of the three most recent data points) and the stalagmite Itzamna became ‘dormant’ for 20 thousand years. This declining precipitation trend coincides with a reduction in Itzamna speleothem growth thus reinforcing the isotopic evidence for precipitation declines (Fig. S3). A long-term shift from high to low precipitation regimes at ~24.5 ka BP is also suggested by other northern and, interestingly, southern hemisphere climate records. In the northern hemisphere, this climate transition occurs at ~24.6 ka BP in the speleothem monsoon δ^18^O record from Hulu Cave in Eastern China (32°30′N, 119°10′E)^4^ and at ~24.7 ka BP in an ocean sediment total reflectance L* record from the northeastern Arabian Sea (23°07.34′N, 66°29.84′E)^8^ (Fig. 3). In the southern hemisphere, a similar climate transition occurs at ~24.4 ka BP in a speleothem δ^18^O record from Pacupahuain Cave in the central Peruvian Andes (11.24°S, 75.82°W)^7^ and at ~24.8 ka BP in a speleothem δ^18^O record from Paraíso Cave in the Amazon basin (4°4′S, 55°27′W)^15﻿^ (Fig. 4). The Cariaco Basin sediment total reflectance record (10°40.69′N, 64º58.29′W)^8^, interpreted to reflect the mean position of the ITCZ, also shows a transition from low to high reflectance values at the time (Fig. 3). Together, these observations would suggest that the coeval change in the Cariaco Basin sediment reflectance record probably reflects a decrease in the intensity of tropical convective activity and not a significant shift in the ITCZ position as would be observed over stadial-to-interstadial transitions (Figs 3 and 4). We want to point out that the observation of synchronous and in-phase precipitation reduction in the American tropics during stadial 2 does not contradict evidence for an anti-phase precipitation relationship on stadial-interstadial time scales, which is supported by a number of studies^7, 8, 14, 15^. The Itzamna record spans stadial 2 and thus we cannot infer precipitation conditions during the following or preceding interstadial time intervals and detect the anti-phase relationship during full D-O events that is revealed by tropical paleoclimate records.

The hydrological response across the American tropics occurs during the onset and duration of H2. Magnetic susceptibility and mineralogical records from the high-resolution (20 cm/1000 year) Iberian Margin Core SU8118, one of the best-dated sediment records of H2, suggest two IRD depositional phases centered at 23.5 ± 0.2 ka BP and 25 ± 2 ka BP^13^, in agreement with other assessments of H2 based on sediment cores from the North Atlantic^12^ (Fig. 2). These depositional phases occurred in association with cooling and advection of low-salinity arctic water masses in the subtropical Atlantic, as suggested by alkenone records from core SU8118^13^.

Progressive precipitation decline in the YP (this study), Central America^6, 55^, India and South East Asia, coeval with precipitation reduction in Perú^7^ and the Amazon Basin^15^, associated with H2, are consistent with model results of AMOC slowdown. Particularly, hosing experiments based on atmosphere–ocean general circulation models suggest that freshening of the North Atlantic and the ensuing AMOC slowdown and atmospheric cooling would cause significant precipitation reduction in the American tropics, including the Caribbean and Amazon basin^16, 17^. Notably, the magnitude of precipitation decline during the most intense rainfall reduction events suggested by the Itzamna record of ~−50% is consistent with model results of peak precipitation reduction in the Caribbean region associated with AMOC slowdown^16^.

We suggest that extreme drought conditions in the YP after ~24.5 ka BP was an inevitable pattern that followed high regional precipitation in the Caribbean and warm ocean anomalies in the North Atlantic, the latter possibly related to a positive AMO phase. North Atlantic warming and freshening combined would trigger massive iceberg discharges into the North Atlantic (i.e. H2) and the resulting meridional propagation of cooling via advection and air-sea interactions^19^ would then cause a reduction in low latitude rainfall^16^. The North Atlantic low latitude precipitation reduction by the end of S2 was intense enough to have likely played a counteracting role to AMOC slowdown. North Atlantic high salinity induced by intense low latitude precipitation deficits, as the Itzamna precipitation record suggests, would propagate to the high latitudes, increase upper ocean convection, and help resume AMOC, as proposed by previous studies^57–59^. A recent study that investigates the northward flow of warm water through the Florida Strait, from benthic foraminiferal δ^18^O records from sediment cores, does not find evidence of significant shifts in the Atlantic overturning circulation during H2^60^. The implication of these findings by ref. 60 is that precipitation reductions in the YP and presumably in the northern Caribbean region resulting from the meridional propagation of cooling during H2 may have helped counteract AMOC slowdown during this time.

The Arabian Sea and Cariaco Basin sediment reflectance values during S2 are the lowest of all stadial events, thus suggesting to be the wettest of all stadial events, and comparable to values observed during interstadials 9, 18 and 20 within these records. These observations suggest that the ITCZ was not particularly displaced south during S2. The evidence presented thus far implies that cooling associated with Heinrich events can significantly impact tropical and subtropical hydroclimate during stadials without involving a shift in the mean position of the ITCZ. In the specific case of S2, Atlantic cooling could have induced lower precipitation across the tropical Americas by decreasing the atmosphere’s moisture holding capacity and, by decreasing the intensity of tropical convection^16^. Tropical Atlantic cyclogenesis was also likely reduced during the last glacial and particularly during cool stadial episodes, when tropical convection and SSTs in the main developmental region were reduced^61^. Precipitation reductions in the Caribbean region during S2 could have thus been amplified by a lower potential of tropical cyclones to transfer moisture to higher latitudes^46, 62^.

This work provides new high-resolution stalagmite δ^18^O and δ^18^O-derived precipitation records that help characterize the magnitude and frequency of Caribbean hydroclimate variability on interannual and multidecadal time scales during a stadial event. Our study offers new evidence that precipitation across the American tropics declined progressively in close association with the onset and duration of Heinrich event 2. The geographical pattern and magnitude of tropical hydrological change during stadial 2 suggested by paleoclimate records, furthermore, are consistent with model results of AMOC slowdown. We propose that increased salinity in the Caribbean region driven by a precipitation deficit resulting from North Atlantic cooling associated with H2 played a crucial role in helping counteract AMOC slowdown. In light of the evidence provided, we suggest that tropical hydroclimate does not only respond to but also influences AMOC circulation during glacial intervals, in agreement with previous studies^57–59^.

Lastly, we suggest that the Itzamna stalagmite precipitation estimates be considered as preliminary and not as an end result. We anticipate that modeling studies will help expand our understanding of the amount effect and the isotopic composition of precipitation during the last glacial and thus help test our precipitation inferences. The long-term monitoring of precipitation we are conducting will reveal the robustness of the link between tropical precipitation amount and precipitation δ^18^O on interannual time scales revealed by models^22^, while helping us understand the factors that control this relationship and those that may disrupt it, such as tropical cyclones^63^. Speleothems from tropical regions may represent our best chance to reconstruct low latitude precipitation variability and its sensitivity to shifts in climate boundary conditions (e.g. greenhouse gases) and processes internal to the climate system (e.g. AMOC).

## Methods

### U-Th dating

Calcite powders weighing from 0.1 to 0.15 g were used for U-Th chemistry^24^. Uranium and thorium isotopic measurements were conducted on a multi-collector inductively coupled plasma mass spectrometer (MC-ICP-MS), Thermo Fisher Neptune, at the High-Precision Mass Spectrometry and Environment Change Laboratory (HISPEC), Department of Geosciences, National Taiwan University^26^. A triple-spike, ^229^Th-^233^U-^236^U, isotope dilution method was employed to correct for mass bias and determine all uranium and thorium isotopic and concentration values in an off-line data reduction process developed by ref. 64. All errors of isotopic data and dates given (Table S1) are two standard deviations (Supplementary materials Fig. S7). We developed the chronology of this section based 2000 Montecarlo simulations using the COPRA software^27^ (Table S2).

Calcite δ^18^O analyses were performed at the University of Massachusetts using an on-line carbonate preparation system linked to a Finnigan Delta Plus XL isotope ratio mass spectrometer. Reproducibility of the standard materials is better than ± 0.1‰. Values are reported relative to the V-PDB standard.

### Cave Monitoring

We monitored relative humidity daily in two sectors of Río Secreto including at the stalagmite Itzamna collection site during two full years, from June 2014 to June 2016 (n = 5000 measurements using HOBO instruments). Relative humidity of 100% was recorded throughout this time. The cave floor is currently below the water table and thus provides a constant moisture source that is particularly retained in cave chambers. We recognize that these conditions were likely different during the last glacial when sea level and thus the water table in the cave were much lower than today. Cave relative humidity during the last glacial may have remained, however, close to 100% because of lower evaporation resulting from global cooling. In addition, we have monitored caves with no standing water inside in the northwest most arid region of the YP^53^ and found relative humidity at or near saturation levels. We monitored cave temperature inside the chamber of Itzamna, known as Laberinto del Fauno, for 1.5 years from June 2015 to October 2016 (n > 1787 measurements using HOBO instruments). Measured cave air temperature inside this chamber was 23 ± 1 °C thus reflecting 3 °C colder mean temperature than the annual mean air temperature in Playa del Carmen of 26 °C^21^. In June 2014 we initiated a weekly collection of drip waters from different stalactites in various chambers within the cave system. Results from 163 dripwater measurements taken between the year 2014 and 2016 indicate an average drip water δ^18^O composition of −3.7 ± 1.0‰ in Laberinto del Fauno. The observed drip δ^18^O variability was in tune with the seasonal cycle of precipitation consistent with a relatively short mixing time of drips^23^. The mean drip water δ^18^O value (−3.7 ± 1.0‰) is very similar to the annual amount weighted δ^18^O value (−4‰) of rainfall in Playa del Carmen during this interval of time (n = 13 months). Modern dripwater temperature in this chamber is 23 °C, based on 1.2 years of weekly measurements of two drips (n = 62 weeks). These observations suggest that it is unlikely for evaporation to significantly influence dripwater geochemistry at these sites. Measured Laberinto del Fauno dripwater and groundwater temperatures are similar to the cave air temperature (23 ± 1 °C). Similar cave air, dripwater and groundwater temperatures that are 3 °C colder than the mean annual surface air temperature suggests that dripwater temperature reflects cave air temperature and this in turn groundwater temperature because groundwater is present year round in the Rio Secreto cave system.

### Amount effect

Instrumental records of precipitation amount and rainfall δ^18^O, and modeling studies, indicate that there is an amount effect on seasonal and interannual time scales in the Yucatan Peninsula^22, 23^. We have previously characterized the seasonal amount effect between precipitation amount *(P)* and precipitation δ^18^O (*dP*) by monitoring rainfall amount and rainfall δ^18^O in the city of Cancún in the year 2012 and in Río Secreto in 2014^23^. The amount effect on seasonal time scales in the YP is represented by the slope *δP*/*ΔP* = −0.0137 ± 0.0031‰/mm (80% confidence interval, CI, r = 0.87). The amount effect is statistically similar (80% CI) to that recorded in Veracruz, México (−0.0125‰ per mm), in San Salvador, El Salvador (−0.0124‰ per mm)^23, 65^, Havana, Cuba (−0.0147‰ per mm) and Tuscaloosa, Alabama (−0.0132‰ per mm) (GNIP, IAEA). We currently have no means to assess the value of the amount effect in the YP during the last glacial time interval. The similar amount effect slope found in the northern Caribbean and Gulf of Mexico regions suggest that this parameter is relatively stable across different climate regimes. In order to include a measure of uncertainty regarding the amount effect, this study uses the full confidence interval of the amount effect slope measured in the northeast of the YP to calculate precipitation changes. The slope range (i.e. −0.0168 and −0.0106‰ per mm) well exceeds values observed in northern Caribbean and Gulf of Mexico locations today.

### Expected equilibrium stalagmite δ^18^O values

Oxygen isotope data from Río Secreto indicate that dripwater δ^18^O composition closely reflects the annual amount-weighted δ^18^O composition of rainfall^23^. Applying the equation by Tremaine *et al*.^32^, we calculate the expected stalagmite δ^18^O composition precipitated under oxygen isotopic equilibrium conditions with cave dripwater annual temperature (23 °C), by using the local amount effect relationship characterized instrumentally (Y = −0.0137x − 0.9525), and 5-year monthly averages of precipitation amount (1998–2003, 1465 ± 280 mm/yr). The equilibrium calculations yield a stalagmite δ^18^O composition of −4.1‰, which is within the variability range of the most recent section of Itzamna (−4.8 ± 1.3‰, 2 SD) spanning the Preclassic Period of Maya history (BCE 1037 to CE 397)^23^. The Itzamna record we present in this study has a mean δ^18^O composition of −3.1‰ ± 1.8‰ (2 SD), that is, 1.7‰ more positive than the mean δ^18^O composition of the younger section (Fig. S8). Comparisons of Holocene and Last glacial speleothem δ^18^O records would suggest that the YP was drier during the last glacial relative to the Holocene, even when considering a last glacial positive shift in ocean δ^18^O of 1‰ due to the expansion of high latitude ice sheets^66^. Glacial temperature and rainfall δ^18^O, however, also need to be taken into account when comparing Holocene and glacial stalagmite δ^18^O values.

### Principle for quantitative precipitation estimates

Precipitation δ^18^O is controlled by precipitation amount on seasonal and interannual time scales in the YP, therefore, if it rains the same amount in five consecutive years, the precipitation δ^18^O composition (amount weighted) is expected to remain unchanged. If precipitation amount changes on one given year, then precipitation δ^18^O would change according to the amount effect relationship. Precipitation estimates from stalagmite δ^18^O represent the average precipitation amount of one, two or the number of years our sample integrates. Depending on the site, seasonal dripwater δ^18^O could conceivably integrate the annual precipitation isotopic signal, as we observe in some drips from Rio Secreto, in which case, stalagmite δ^18^O would closely represent an annual precipitation δ^18^O signal integration (albeit with some delay), even when it is sampled on seasonal resolution. Because stalagmite δ^18^O per mil shifts are assumed to reflect similar shifts in precipitation δ^18^O, interannually, we can then estimate precipitation amount shifts from stalagmite δ^18^O anomalies calculated relative to a reference point. We maintain the local modern annual precipitation cycle but shift annual precipitation amount and therefore precipitation δ^18^O so that annual mean precipitation δ^18^O changes in accordance to the interannual δ^18^O precipitation anomalies reflected by the stalagmite^30^. Seasonality may produce precipitation amount biases on both directions, positive and negative, using this method. Summer precipitation to total annual precipitation on typical years today in the northeast YP range from a minimum of 60% to a maximum of 70% summer precipitation (Fig. S2). We estimated how much these shifts in seasonality associated with the same amount of annual precipitation would affect our precipitation inferences. The potential seasonal precipitation bias in our estimates is ±8%, within the range of 55 to 70% summer contribution to annual precipitation; a 5% larger seasonality than observed today.

### Quantification of precipitation amount from Itzamna during the last glacial

We use the 5-year annual mean of precipitation (1998–2003) in Playa del Carmen of 1465 mm as the reference precipitation value for our estimates of precipitation change^23^. The last glacial equilibrium δ^18^O composition of Itzamna (δ^18^O_c-PDB_) for this reference value is calculated with the paleotemperature equation by ref. 32:

$${\rm{t}}(^\circ {\rm{C}})=1000/(((1000\ast \,\mathrm{Ln}\,{\rm{\alpha }})+24.6)/16.1)$$

where α = (1000 + δ^18^O_c-VSMOW_)/(1000 + δ^18^O_w-VSMOW_) and δ^18^O_cVSMOW_ = 30.92 + 1.03092*(δ^18^O_c-PDB_).

Here the last glacial cave dripwater δ^18^O composition (δ^18^O_w-VSMOW_) in Río Secreto is determined from assuming the annual δ^18^O composition of precipitation (amount-weighted) associated with 1465mm/yr (reference value). This assumption is supported by instrumental monitoring data as detailed above^23^. The last glacial annual δ^18^O composition of precipitation (amount-weighted) was determined from adjusting a 1.6‰ higher last glacial annual precipitation δ^18^O value relative to today, as suggested by global circulation models GCMs^67, 68^. We want to point out that because few studies exist that investigate shifts in precipitation δ^18^O during glacial intervals, it is difficult to assess how certain/uncertain this adjustment of precipitation δ^18^O may be, although a minimum of ~1.2‰ positive shift in precipitation is expected as a result of the last glacial shift in ocean δ^18^O due to expansion of the continental ice sheets^66^. Evidently, interpretations of speleothem δ^18^O records from tropical regions would greatly benefit from increasing development of studies focused on isotope-enabled GCMs that target glacial conditions.

The S2 temperature of dripwaters (t) was calculated by subtracting a tropical glacial cooling of 3–5 °C that is suggested by seven Atlantic tropical SST records^31^ from the modern mean annual dripwater temperature of 23 °C. We note that, this Holocene-to-last glacial SST range also applies to the Arabian Sea, Indian Ocean and tropical Pacific^31^. We consider this 2 °C temperature range as uncertainty associated with our estimates of last glacial cave temperature. This yields a final dripwater temperature estimate ranging from 18 °C to 20 °C. The resulting equilibrium δ^18^O composition of calcite associated with last glacial conditions and 1465 mm/year ranged from −1.4‰ at 18 °C to −1.7‰ at 20 °C.

Stalagmite δ^18^O anomalies were then calculated in reference to these two isotopic equilibrium values. Positive and negative δ^18^O anomalies would therefore indicate lower and higher precipitation relative to 1465 mm, respectively. Precipitation percent changes were then calculated based on the full confidence interval of the slope of the relationship between the amount of precipitation and precipitation δ^18^O (*δP*/*ΔP* = −0.0137 ± 0.0031‰ per mm), equivalent to the slopes −0.0106‰/mm and −0.0168‰/mm. As expected, the steepest slope will yield the lowest precipitation change associated to a given δ^18^O anomaly. As an example, a positive stalagmite anomaly of + 1‰ would be explained by shifting precipitation amount and therefore the annual cycle of precipitation δ^18^O (last glacial adjusted) by −55% and −35%, using the slopes −0.0106‰/mm and −0.0168‰/mm, respectively. We note that the precipitation estimates associated with a stalagmite δ^18^O anomaly, for instance of +1‰, depend on the reference value. If we increased 50% the reference value 1465 mm to 2194.5 mm, then a +1‰ anomaly would be explained by annual precipitation amount reductions of −38% and −24% using the slopes −0.0106‰ per mm and −0.0168‰ per mm, respectively.

We used the paleotemperature equation by Tremaine *et al*.^32^ because this equation applies to a broad range of temperature and cave environments. We explored the alternative paleotemperature equation by Kim and O’Neil^69^ and found that precipitation estimates not based on the steepest slope (−0.0168‰/mm) yielded precipitation declines in excess of 100%. Precipitation declines >100% would not support this equation, or suggest that the amount effect was similar or higher than *−*0.0168‰ per mm during S2. Alternatively, excess precipitation declines could indicate underestimation of the last glacial precipitation δ^18^O shift, underestimation of the magnitude of cooling, and/or calcite disequilibrium precipitation during peak drought. The last glacial precipitation δ^18^O shift that would explain the most positive stalagmite δ^18^O value (0.1‰) without resulting in precipitation declines in excess of 100% over the temperature and amount effect conditions applied is 2.4‰, which is 0.8‰ more positive than the value suggested by climate model results (1.6‰)^67, 68^ and ~1.2‰ more positive than explained by a shift in ocean δ^18^O associated with the expansion of the continental ice sheets^66^. A larger precipitation δ^18^O adjustment is therefore unsupported by available model results and paleoclimate proxy records. Examination of available last glacial subtropical and tropical Atlantic sea surface temperature records ^70, 71^, on the other hand, does not suggest Caribbean sea glacial cooling larger than 5 °C. Lastly, we cannot discard disequilibrium isotopic conditions during peak drought events induced by decreasing cave relative humidity. The atmospheric demand of moisture and thus evaporation, however, were lower during the last glacial due to regional cooling^61^, which would have helped maintain water vapor saturation conditions inside the cave.

Based on the available evidence, we find stronger support for applying the paleotemperature equation by Tremaine *et al*.^32^, which applies to a broad range of cave temperatures and environments similar to Rio Secreto, and does not produce precipitation declines in excess of 100% across the range of temperatures and amount effect slopes applied in this study. Additional precipitation records and the long-term monitoring we are currently conducting in Rio Secreto, involving farmed calcite, will provide more definite support for our chosen paleotemperature equation.

### Assessment of influence of chronological uncertainty on spectral analysis

In order to assess chronological uncertainty effects on our spectral results, we expand and contract the time scale over periods of interest and determine how this affects the duration of the dominant periods suggested by the spectral analysis (Fig. 2A). Specifically, the period of variability that may reflect ENSO variability, centered at ~8 years, is particularly strong over a 700-year time interval between 24.7–25.4 kyr BP. We thus expand and contract the time scale over this time interval by applying the average radiometric age uncertainty (±132 years) and then determine how the dominant period of variability of 8 years would be affected. This analysis yields a dominant period of 9.5 and 6.5 years on the expanded and contracted time scales, respectively. Chronological uncertainties, therefore, translate into a dominant period of variability of 8 ± 1.5 years. Regarding multidecadal time scales and the periods of variability centered at 32 and 64 years, we use the entire interval of 1542 years represented by the spectral analysis plot and expand and contract this time scale by the maximum age uncertainty of ±202 years. This assessment yields periods of variability of 32 ± 4 years and 64 ± 8 years, thus suggesting that chronological uncertainties have little influence on the duration of the dominant periods of variability suggested by our spectral analysis.

## Electronic supplementary material

SOM

Table S1

Table S2

Table S3
