# Supplementary material for: Synchronous precipitation reduction in the American Tropics associated with Heinrich 2
Source: Sci Rep. 2017 Sep 11;7:11216. doi: 10.1038/s41598-017-11742-8 (PMC5593979; doi:10.1038/s41598-017-11742-8)
Supplement: Supplementary file 1 — SOM [file 41598_2017_11742_MOESM1_ESM.pdf]

Synchronous precipitation reduction in the American Tropics associated with Heinrich 2

Martín Medina-Elizalde, Stephen J. Burns, Josué Polanco-Martinez, Fernanda Lases-Hernández, Raymond Bradley, Hao-Cheng Wang, Chuan-Chou Shen.

Supplementary Information

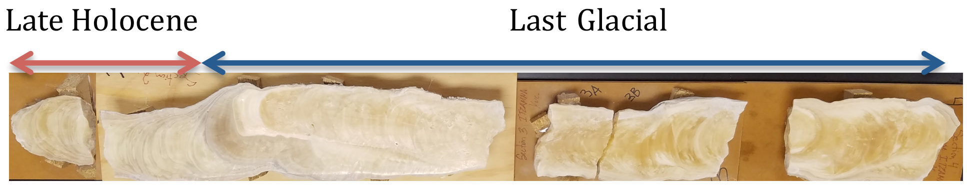

Figure S1. Picture of the Itzamna specimen. This specimen spans the time interval between 26.4 and 23.2 ka BP (Last glacial) and between BCE 1037 and CE 398 (Late Holocene).

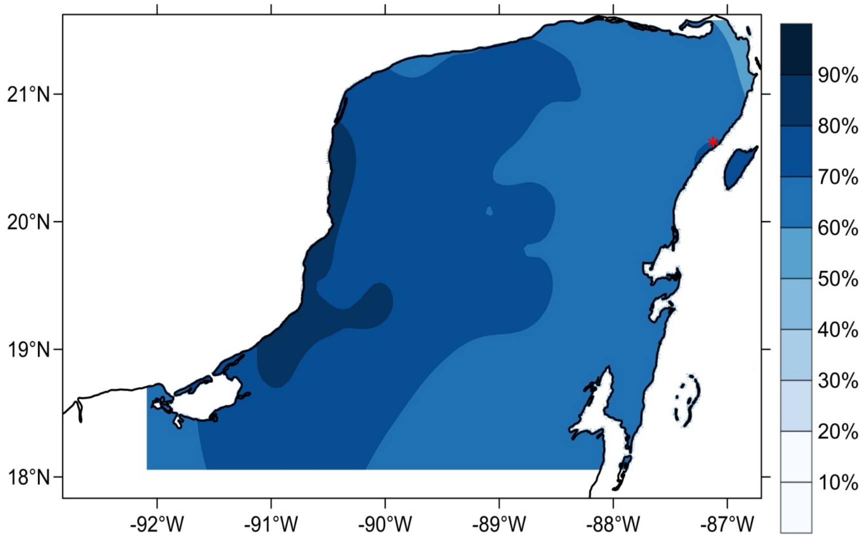

Figure S2 Percent contribution of summer precipitation (June-October) to annual precipitation in the Yucatan Peninsula based on instrumental data from 1926-2011 [1].

Location of the cave is shown (red asterisk). The map was constructed using the software Surfer v.12 by Goldensoftware (http://www.goldensoftware.com/products/surfer).

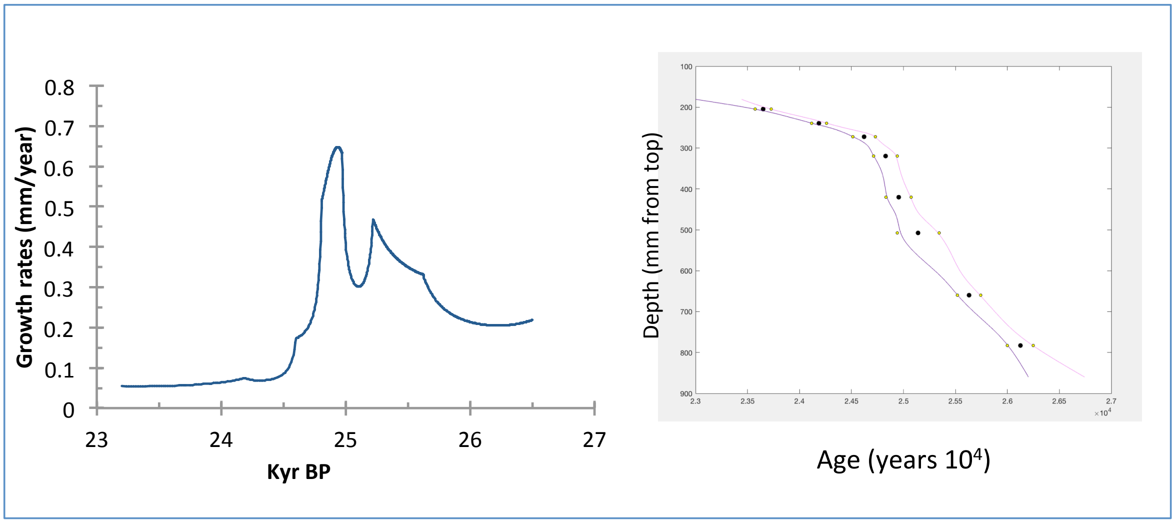

Figure S3. Stalagmite Itzamna growth rates in mm/year (left panel) and age model at a 95% confidence interval (right panel) based on 2000 montecarlo-style simulations using the Software COPRA[2]. The stalagmite achieved peak growth rates at ~ 25 ka BP and lowest rates when it ceased growing at ~23.2 ka BP.

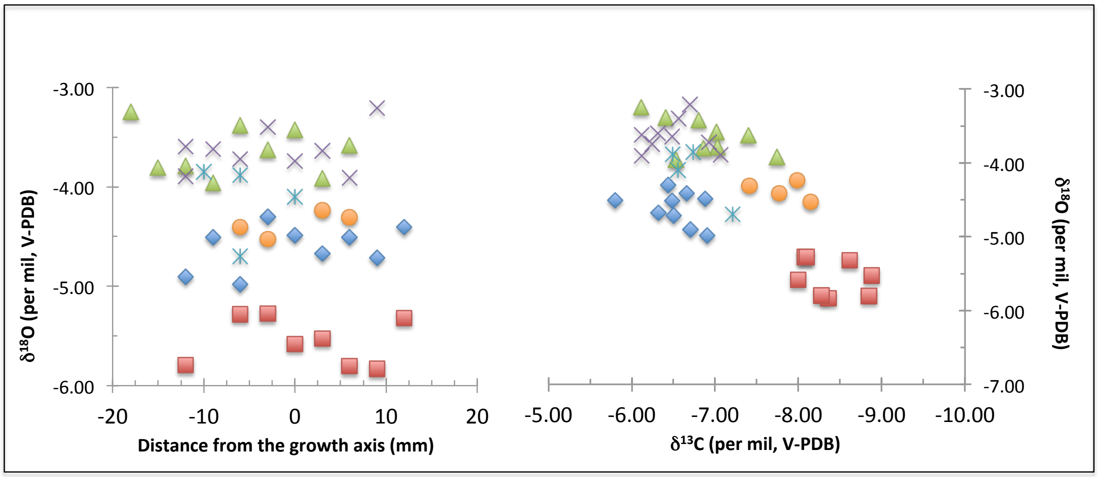

Figure S4. Hendy test for the speleothem Itzamna[3]. (Left) $\delta^{18}O$ profiles along the same growth layer for 6 different growth layers (different colors). (Right) $\delta^{18}O$ versus $\delta^{13}C$ along the same growth layer as in left panel, suggesting no kinetic isotopic fractionation. Sample

depths are 45, 60, 86, 170, 314 and 800 mm from the top, including the Holocene and Last Glacial intervals of growth.

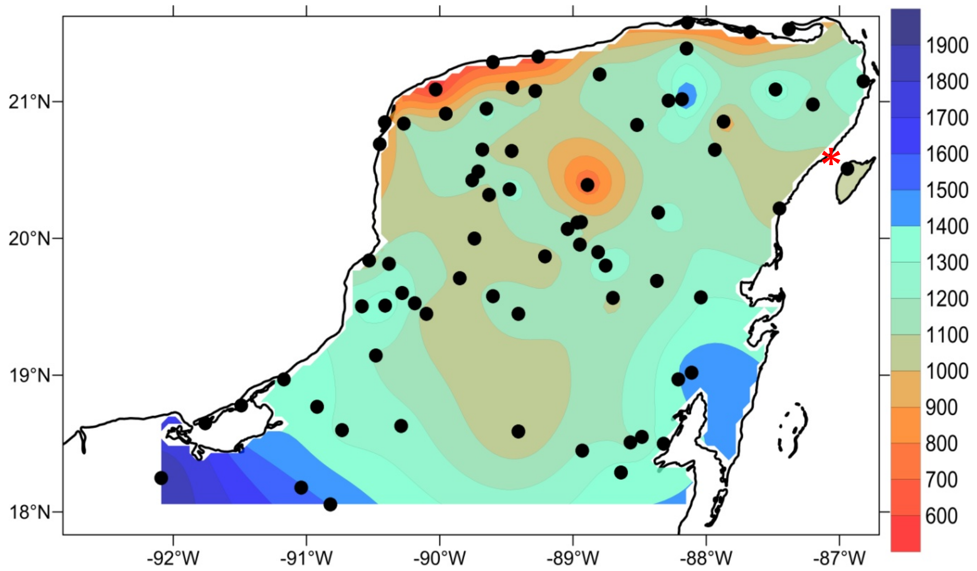

Figure S5. Annual precipitation in the Yucatan Peninsula (mm/year) based on data from 76 meteorological stations with a record of between 11 and 84 years spanning the time interval between 1926-2011 [4]. Cave location is shown (red asterisk). The map was constructed using Surfer v.12 by Goldensoftware (http://www.goldensoftware.com/products/surfer).

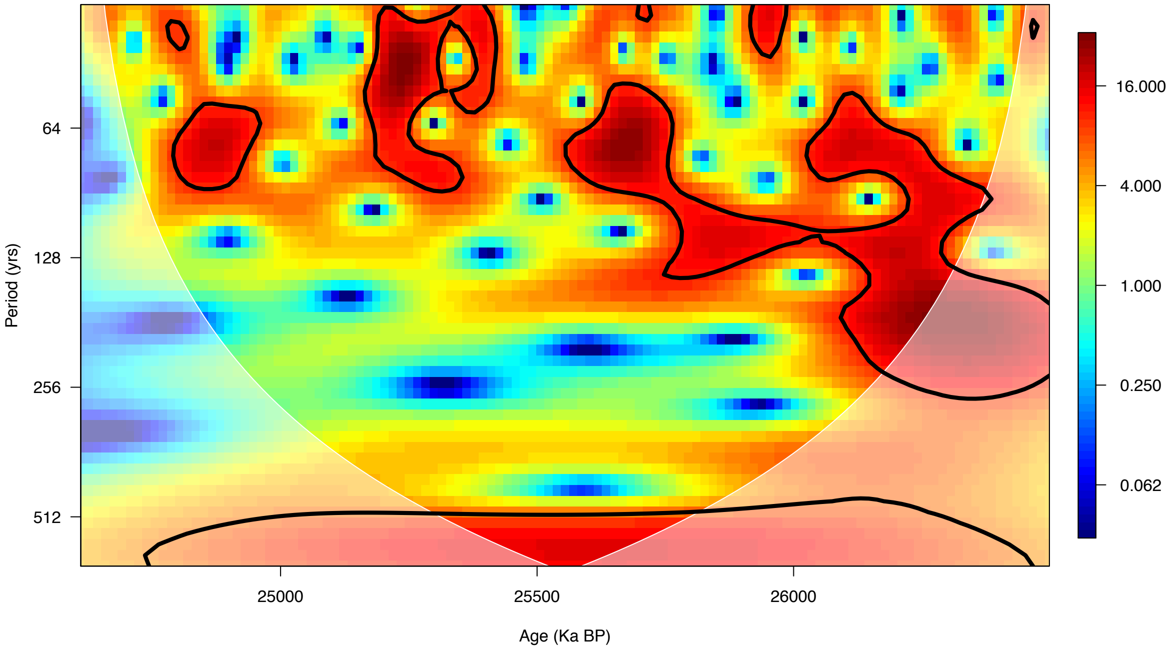

Figure S6. Wavelet spectral power analysis of the Itzamna $\delta^{18}O$ record based on data interpolated at the lowest time resolution of the record of 16 years observed between 23.2 and 24.5 ka BP. The area outside the white envelop indicates the cone of influence where the edge effects of the wavelet transform and uncertainties become important (CI=95%). Wavelet spectral power is based on methods by refs.[5,6], as implemented in the R package "biwavelet"[7].

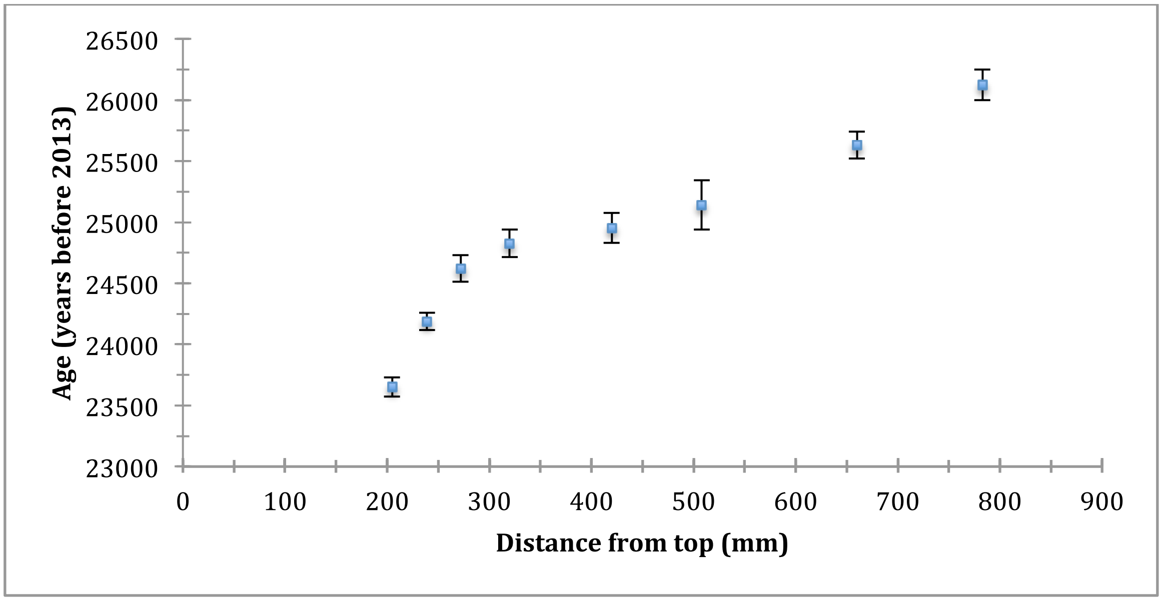

Figure S7. U-Th radiometric dates as a function of distance from the top of stalagmite. Age uncertainties represent 2 standard deviations. The chronology of this section is based on a piecewise-linear model to account for non-linearity in stalagmite growth.

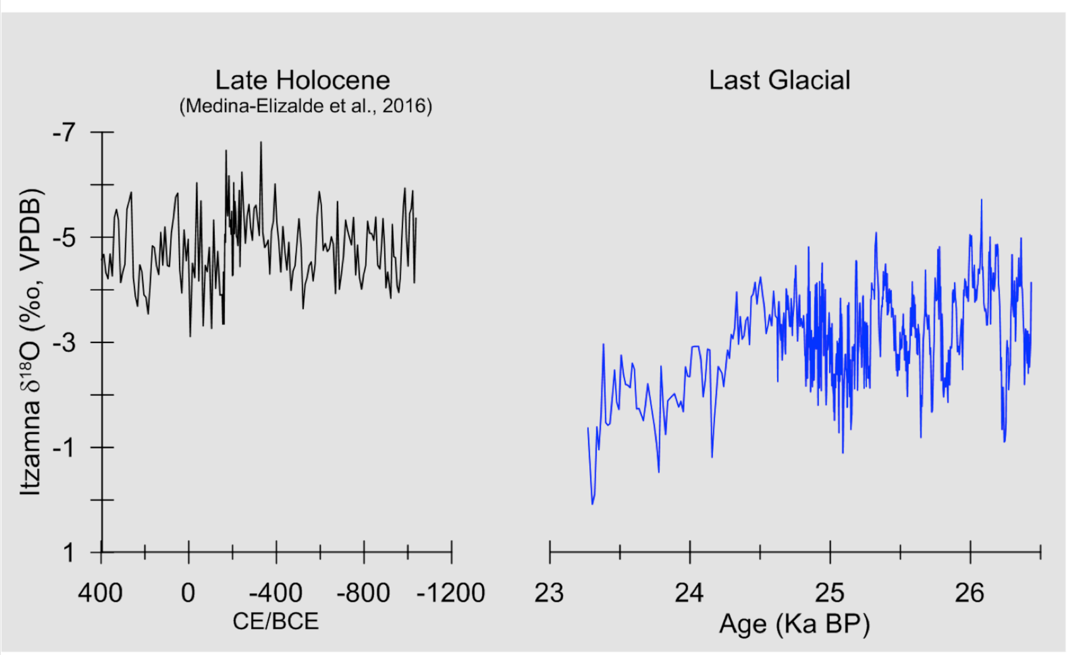

Figure S8. Itzamna stalagmite $\delta^{18}O$ records spanning the interval between BCE 1037 and CE 397 [8], and the interval between 26.4 and 23.2 ka BP (this study).

References

1 CONAGUA. *Servicio meteorológico nacional, México (available at: http://smn.cna.gob.mx/)* (2012).

2 Breitenbach, S. F. M. *et al.* Constructing Proxy Records from Age models (COPRA). *Clim Past* **8**, 1765-1779 (2012).

3 Hendy, C. H. The isotopic geochemistry of speleothems—I. The calculation of the effects of different modes of formation on the isotopic composition of speleothems and their applicability as palaeoclimatic indicators. *Geochim Cosmochim Ac* **35**, 801-824 (1971).

4 CONAGUA. *Normales Climatológicas*, <http://smn.cna.gob.mx> (2012).

5 Liu, Y., San Liang, X. & Weisberg, R. H. Rectification of the bias in the wavelet power spectrum. *Journal of Atmospheric and Oceanic Technology* **24**, 2093-2102 (2007).

6 Torrence, C. & Compo, G. P. A practical guide to wavelet analysis. *Bulletin of the American Meteorological Society* **79**, 61-78 (1998).

7 R Core Team (2013) R: A language and environment for statistical computing. R Foundation for Statistical Computing, Vienna, Austria. URL http://www.R-project.org/" (2013).

8 Medina-Elizalde, M. *et al.* High-resolution speleothem record of precipitation from the Yucatan Peninsula spanning the Maya Preclassic Period. *Global Planet Change* **138**, 93-102 (2016).
